# Latest observed volumetric MRI response and lesion level local control after gamma knife radiosurgery for breast cancer brain metastases

**DOI:** 10.3389/fmed.2026.1998918

**Published:** 2026-10-28

**Authors:** Sijun Diao, Tao Huang, Zhongyu Li, Shihong Zhu, Fujun Liu, Xiyue Lu, Huiyang Luo, Jing Chen

**Affiliations:** 1 Department of Neurosurgery, West China Hospital, West China Medical School, Sichuan University, Chengdu, China; 2 Department of Anesthesiology, West China Hospital, West China Medical School, Sichuan University, Chengdu, China

**Keywords:** breast cancer brain metastases, gamma knife radiosurgery, local control, serial MRI, treatment response assessment, volumetric response

## Abstract

**Background:**

Serial magnetic resonance imaging (MRI) reveals heterogeneous lesion responses after stereotactic radiosurgery beyond binary local-control status. We characterized latest-observed volumetric response categories after Gamma Knife radiosurgery (GKRS) for breast cancer brain metastases and examined their concordance with 12-month local control (LC_12_m).

**Methods:**

This retrospective study included 80 patients, 182 lesions, and 819 MRI records. LC_12_m incorporated earlier local failure or assessment within a 10–14-month window. Four response categories compared the latest eligible whole-tumor (WT) volume with baseline. Baseline imaging and dose correlates of failure were evaluated using variational Bayesian mixed-effects logistic regression and patient-clustered generalized estimating equations (GEE). Early-landmark analyses explored associations with subsequent failure.

**Results:**

LC_12_m was evaluable in 105/182 lesions (70 controlled, 35 failed). Among 154 response-classifiable lesions, 24 (15.6%) showed complete response, 56 (36.4%) partial regression, 48 (31.2%) stable volume, and 26 (16.9%) enlargement. Enlargement was strongly associated with failure (GEE odds ratio 16.797, 95% confidence interval 3.398–83.033). Contemporaneous concordance measures were 54.3% sensitivity, 97.1% specificity, 90.5% positive predictive value, and 81.0% negative predictive value; 97/105 category assessments occurred within the endpoint window. Early-landmark analyses were inconclusive. In 102 complete cases, variational Bayesian odds ratios were 1.888 [95% credible interval (CrI): 1.079–3.302] per unit increase in ln[1 + WT (cm^3^)], 3.071 (1.520–6.203) for edema, and 0.760 (0.697–0.829) per 10-Gy increase in BED_10_. GEE estimates were directionally consistent but did not reach statistical significance. Same-model, same-prior Markov chain Monte Carlo analysis supported the inverse association between BED_10_ and local failure, whereas evidence for the WT and edema associations remained inconclusive.

**Conclusions:**

Serial volumetric MRI and latest-observed response categories complement binary local-control assessment by describing heterogeneous lesion-level responses after GKRS. Enlargement showed strong contemporaneous concordance with LC_12_m, with overlapping definitions and assessment times limiting claims of independent prediction. Baseline imaging and BED_10_ associations were model-dependent. External validation is needed to establish the framework's added clinical value.

## 1 Introduction

Brain metastases are a major cause of neurologic morbidity in advanced breast cancer. Intracranial risk varies by molecular subtype and is particularly high in human epidermal growth factor receptor 2 (HER2)-positive and triple-negative disease (1, 2). Prognosis is also shaped by performance status, intracranial disease burden, receptor discordance, and subtype switching (3–5). Modern systemic agents, including tucatinib-based therapy and trastuzumab deruxtecan, have demonstrated clinically meaningful intracranial activity in HER2-positive disease (6, 7); nevertheless, local therapy remains central for patients with limited or clinically actionable brain metastases. Stereotactic radiosurgery (SRS), including Gamma Knife radiosurgery (GKRS), is guideline-supported and can avoid the neurocognitive burden of whole-brain radiotherapy in appropriately selected patients (8–12).

Local failure nevertheless remains clinically relevant after SRS/GKRS. Breast-cancer-specific series have linked recurrence to lesion size and treatment factors, but many reports emphasize patient-level survival or a single binary local-control endpoint (13, 14). Recent subtype-specific SRS cohorts in HER2-positive and triple-negative breast cancer further demonstrate heterogeneous intracranial and lesion-level outcomes (15, 16). This approach does not fully represent lesion-to-lesion heterogeneity: metastases in the same patient may disappear, regress gradually, remain stable, or enlarge after treatment. Dose is also not randomly assigned; prescription is shaped by lesion volume, location, prior treatment, and normal-tissue constraints. Classical dose-escalation studies and lesion-level analyses have consistently linked prescribed dose and tumor volume with local control (17, 18), while multifraction SRS has been associated with improved local control and lower radionecrosis risk for larger lesions in comparative cohorts (19). A lesion-level analysis that accounts for clustering within patients is therefore needed to evaluate baseline imaging phenotype and treatment intensity together.

Serial MRI provides a complementary view of treatment response. The Response Assessment in Neuro-Oncology Brain Metastases (RANO-BM) criteria provide a standardized framework, but one-dimensional assessment can be difficult for small or irregular post-SRS lesions, and transient enlargement may represent tumor, radiation injury, or mixed treatment effect (20–23). Volumetric categories adapt these response concepts but remain exploratory; a latest-volume category does not capture the full trajectory.

We therefore evaluated serial volumetric MRI measurements and latest-observed response categories after GKRS for BCBM, assessed their concordance with 12-month lesion-level local control, and examined baseline imaging and dose correlates of failure. Early-landmark analyses evaluated subsequent failure.

## 2 Materials and methods

### 2.1 Study design and cohort

This retrospective study was conducted at West China Hospital, Sichuan University. Consecutive patients who underwent GKRS between April 2021 and August 2025 were retrospectively screened, and follow-up data were collected through August 2026. The study was conducted in accordance with the Declaration of Helsinki and was approved by the Biomedical Ethics Review Committee of West China Hospital, Sichuan University, on 6 August 2026 (approval No. 2026 Review No. 1838). The requirement for written informed consent was waived because of the retrospective design and the use of de-identified clinical and imaging data, and the approval covered the publication of anonymized MRI images. Patients had pathologically confirmed breast cancer, radiologically confirmed brain metastases, and underwent GKRS according to institutional treatment practice. In accordance with the institutional indication for GKRS, all included patients had no more than 10 visible intracranial lesions before the index treatment course, consistent with prospective evidence supporting SRS alone for carefully selected patients with up to 10 brain metastases (24). The study cohort comprised 80 patients and 182 treated lesions. Lesions were the unit for local-control and serial volumetric analyses; patient identifiers were retained to preserve linkage and account for within-patient clustering.

### 2.2 GKRS and dose variables

Treatments were delivered using Gamma Knife Icon and planned in Leksell GammaPlan version 11.1.1. Contrast-enhanced T1-weighted MRI was the principal planning sequence; T2-weighted and fluid-attenuated inversion recovery (FLAIR) images supported boundary and edema assessment. Targets were delineated by an experienced Gamma Knife neurosurgeon and reviewed by the institutional team in accordance with established technical principles for brain-metastasis radiosurgery (25). Session number, prescription or margin dose, prescription isodose line, target volume, and dose summaries were extracted from treatment records and matched to each lesion. BED_10_ was calculated as *D*[1 + *D*/(10*n*)], using *α*/*β* = 10 Gy (26, 27). D was the total mean target dose over the initial treatment course, and *n* was the number of fractions obtained from treatment records. For the 46 lesions treated in two equal fractions, the recorded total D was divided by two to obtain the per-fraction mean dose; the other 136 lesions received one fraction. Subsequent salvage irradiation was not included in the baseline dose metric. EQD2 was BED_10_/1.2. Treatment schemes and calculation parameters are reported in Supplementary Table S9. Four lesions lacked usable dose values and were excluded from dose-dependent analyses.

### 2.3 MRI and segmentation

Baseline and follow-up MRI examinations were acquired on the same 3.0-T GE SIGNA Premier scanner. Contrast-enhanced T1-weighted images had a slice thickness of approximately 1.4 mm at baseline and 1 mm during follow-up. T2-FLAIR images had a slice thickness of 3 mm at baseline and either 3 or 5 mm during follow-up. Baseline contrast-enhanced T1-weighted MRI used gadopentetate dimeglumine 0.1 mmol/kg. All images underwent the same preprocessing pipeline: conversion from Digital Imaging and Communications in Medicine (DICOM) to Nearly Raw Raster Data (NRRD), N4 bias-field correction, resampling to 1-mm isotropic voxels, and intensity normalization. Segmentations were performed in 3D Slicer by two independent readers with 8 and 7 years of neuro-oncologic imaging experience, reviewed by a senior neurosurgeon with 25 years of experience, and resolved by consensus. Serial whole-tumor volumes were delineated using the same anatomical definitions and consensus review procedure.

WT encompassed the entire lesion within its outer boundary, including enhancing solid tissue and internal non-enhancing necrotic or cystic components, while excluding peritumoral edema and normal brain tissue. ET comprised the enhancing solid component within the lesion, excluding non-enhancing necrotic and cystic regions. NET comprised non-enhancing tissue within the tumor boundary, including necrotic and cystic components, while excluding non-enhancing abnormalities outside the tumor boundary. ET and NET were mutually exclusive by anatomical definition. WT was independently delineated manually, and the WT volumes used in the analyses were calculated directly from the WT masks rather than by summing ET and NET volumes. Baseline edema was recorded as present or absent because retrospective FLAIR availability and acquisition heterogeneity did not support consistent quantitative edema volumetry.

Interobserver reproducibility was assessed in 30 lesions from 30 patients, sampled from available independent WT masks across baseline-volume strata and response categories. Absolute-agreement single-measure intraclass correlation coefficients [ICC(A,1)] and Dice coefficients were calculated at baseline and follow-up. Sampling, bootstrap intervals, and handling of independently confirmed zero volumes are detailed in Supplementary Table S7.

### 2.4 Endpoints

LC_12_m was the lesion-level endpoint. Lesions without earlier failure were assessed on contrast-enhanced MRI obtained 10–14 months after index GKRS. The scan closest to 12 months was selected when more than one was available. Earlier qualifying tumor progression or lesion-directed repeat GKRS or surgery for progression constituted local failure at the first qualifying date. Volumetric progression was defined as at least a 30% increase from the prior minimum volume, with an additional absolute increase of at least 0.2 cm^3^ for lesions with baseline volume below 1 cm^3^ (20, 21). The volumetric progression criterion was adapted from Ocaña-Tienda et al. (21). The final endpoint incorporated clinical–imaging review; the volumetric criterion alone did not resolve tumor progression vs. treatment effect. This adapted volumetric criterion is distinct from formal one-dimensional RANO-BM assessment. Inadequate follow-up was classified as non-evaluable for the local-control endpoint. Death or missing MRI without established earlier failure was not coded as local control.

Presumed radiation injury was diagnosed clinically and radiologically when increased enhancement and edema in the irradiated region were followed by stability or regression on serial MRI without additional local treatment to that lesion. Enlargement alone did not establish tumor failure in these cases. Histopathologic confirmation was unavailable. Clinical adjudication did not alter measured WT volumes or volume-based response categories.

Local-control status and presumed radiation injury were assessed by an experienced institutional radiologist using serial MRI and relevant clinical and treatment records and reviewed by a second experienced neurosurgeon. Disagreements were resolved through consensus discussion. Cases remaining indeterminate between tumor progression and treatment effect were classified as non-evaluable for the local-control endpoint.

Only lesions treated during the initial GKRS course were included in the response analysis; newly detected untreated intracranial metastases were excluded. For lesions receiving subsequent lesion-directed salvage treatment, response classification used the latest eligible MRI obtained before salvage. Repeat GKRS or surgery performed for progression of the index lesion constituted local failure, irrespective of subsequent shrinkage. Preplanned stages of the initial GKRS course were considered part of the initial treatment and were not classified as salvage or local failure. Treatment directed at another lesion did not constitute failure of the index lesion. Disappearance required explicit imaging confirmation or a documented zero-volume record.

The latest eligible WT measurement from the nominal 3-, 6-, and 12-month collection, including available pre-salvage observations, was compared with baseline: complete response/disappearance required non-visibility or zero volume; partial regression required a reduction of at least 65%; enlargement required an increase of at least 73%; and stable volume lay between these thresholds. Oft et al. used thresholds of ≥65% volume reduction and ≥72.8% volume increase, derived by applying spherical geometry to the unidimensional RANO-BM response criteria (20, 28). The latter threshold was rounded to 73% in this study. This baseline-referenced categorization describes latest-observed response and is exploratory rather than a validated volumetric response system. Unlike the prior-nadir failure rule, these categories summarize net change from baseline. The two assessments therefore share volume information but differ in reference volume and purpose.

### 2.5 Statistical analysis

Continuous variables were summarized as medians and interquartile ranges and categorical variables as counts and percentages. Baseline characteristics were summarized for the full 80-patient cohort and 182-lesion cohort using variable-specific denominators; smaller denominators reflected missing retrospective documentation rather than additional selection. Missing outcomes were not imputed. The primary model used Bayesian mixed-effects logistic regression fitted by mean-field variational Bayes. The formula included an intercept, a patient random intercept, and three prespecified fixed effects: ln[1 + baseline WT volume (cm^3^)], qualitative edema, and BED10 per 10 Gy. The hierarchical model accounts for within-patient clustering and regularizes estimation with limited events. Odds ratios and 95% posterior credible intervals were the primary summaries. A same-covariate sensitivity analysis used a binomial generalized estimating equation (GEE) with an exchangeable working correlation, patient-level clustering, and robust sandwich covariance. The WT odds ratio refers to a one-unit increase in ln[1 + WT (cm^3^)], not a 1-cm^3^ increase. Model implementation and priors are provided in Supplementary Table S2. Same-prior Hamiltonian Monte Carlo provided a sensitivity check of the variational approximation (Supplementary Table S2).

Dose sensitivity analyses included distribution comparisons, volume-adjusted regression, restricted cubic splines, and EQD2 rescaling (Supplementary Table S3). Response–outcome concordance was assessed using patient-clustered GEE and patient-bootstrap intervals; sensitivity, specificity, PPV, and NPV described contemporaneous agreement (Supplementary Table S4). A 10–14-month restriction assessed timing sensitivity (Supplementary Table S5). Systemic treatments were summarized at GKRS and within the following 3 months. Additional sensitivity analyses added molecular subtype or documented HER2-targeted therapy active at index GKRS to the base model containing WT, edema, and BED_10_, using the same cases for each comparison (Supplementary Tables S2 and S6).

Post hoc 3- and 6-month landmark analyses used MRI at 2–3 and 5–6 months, respectively, and required an evaluable later LC_12_m outcome. Patients had to be alive and lesions free of failure or local salvage at the landmark. Response categories used baseline-referenced volumes available by that date. Wider-window and alternative-contrast analyses are detailed in Supplementary Table S8.

## 3 Results

### 3.1 Cohort and endpoint availability

The cohort comprised 80 patients and 182 treated lesions (Figure 1; Table 1). LC_12_m was evaluable in 105 lesions from 47 patients: 70 were controlled and 35 failed. BED_10_ was available in 178 lesions; 102 had complete model data. Response classification was available in 154 lesions from 67 patients, with 393 dated follow-up WT measurements. The other 28 lesions lacked eligible follow-up volumetry. Of 77 non-evaluable LC_12_m outcomes, 47 occurred in patients who died before 10 months without documented earlier failure and four in patients who died during the assessment window without a qualifying MRI. The remaining 26 lacked adequate window imaging or volumetry, or had unresolved progression vs. treatment effect (Supplementary Table S1).

**Figure 1:**
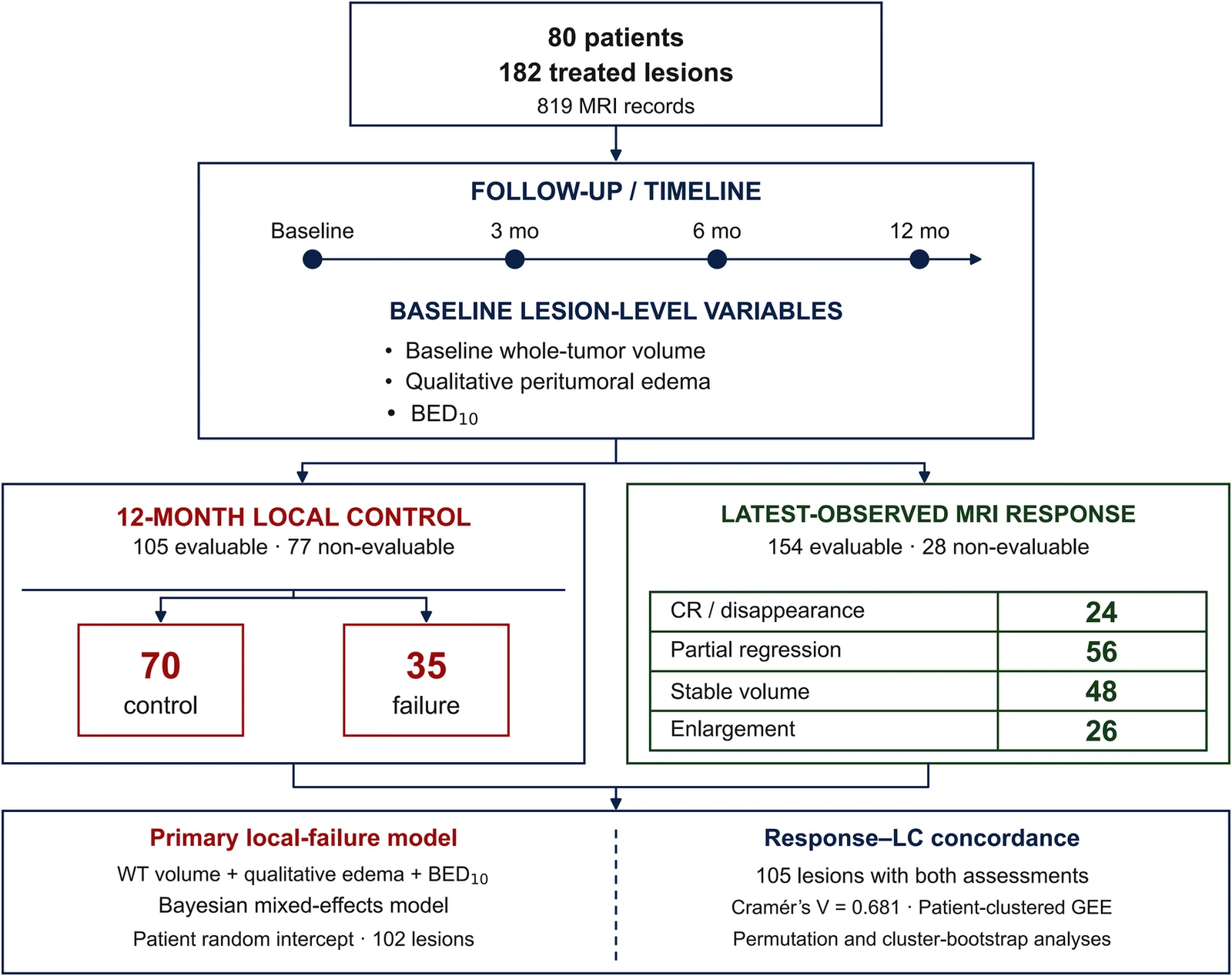
Cohort and analysis populations. LC_12_m was evaluable in 105 of 182 treated lesions, and latest-observed MRI response in 154; the respective non-evaluable groups comprised 77 and 28 lesions. The primary model included 102 lesions after excluding three with missing BED_10_ (two controlled, one failed). GKRS, Gamma Knife radiosurgery; LC, local control; WT, whole tumor.

**Table 1:** Baseline characteristics of patients and GKRS-treated lesions.

Characteristic	Overall
Panel A. Patient-level characteristics (*N* = 80)	
Age at GKRS, years	53 (43–59)
Karnofsky Performance Status, *n* = 54	90 (80–100)
Female sex	80/80 (100%)
Baseline brain metastasis count	2 (1–4)
Molecular subtype, *n*/*N* (%)	
HER2-positive	34/79 (43.0%)
Luminal B	28/79 (35.4%)
Luminal A	2/79 (2.5%)
Triple-negative breast cancer	15/79 (19.0%)
Pathological receptor information, *n*/*N* (%)	
Hormone receptor-positive	19/52 (36.5%)
HER2 IHC 3+	31/52 (59.6%)
Extracranial metastasis	39/54 (72.2%)
Prior whole-brain radiotherapy	13/54 (24.1%)
Prior intracranial surgery	15/54 (27.8%)
Panel B. Lesion- and treatment-level characteristics (*N* = 182)	
Baseline whole-tumor volume, cm^3^	0.380 (0.137–1.414)
Baseline enhancing-tumor volume, cm^3^, *n* = 169	0.376 (0.133–1.303)
Non-enhancing/necrotic component present	58/182 (31.9%)
Qualitative peritumoral edema present	79/144 (54.9%)
Intracranial hemorrhage present	7/130 (5.4%)
Lesion location, *n*/*N* (%)	
Supratentorial	88/130 (67.7%)
Infratentorial	41/130 (31.5%)
Brainstem	1/130 (0.8%)
First-session margin dose, Gy	13.000 (12.000–16.000)
First-session prescription isodose line, %	45.000 (45.000–50.000)
BED_10_, Gy, *n* = 178	57.276 (41.402–83.707)
EQD2 (*α*/*β* = 10 Gy), Gy, *n* = 178	47.730 (34.502–69.756)

Values are presented as median (interquartile range) or *n*/*N* (%). For variables with missing data, denominators are shown in the row label or directly as *n*/*N*. Molecular subtype was available in 79/80 patients and edema in 144/182 lesions; missingness may introduce selection bias and residual confounding. BED_10_, biologically effective dose with *α*/*β* = 10 Gy; EQD2, equivalent dose in 2-Gy fractions; HER2, human epidermal growth factor receptor 2; IHC, immunohistochemistry.

Molecular subtype was recorded in 79 patients: 34 HER2-positive, 28 Luminal B, two Luminal A, and 15 triple-negative. At GKRS, HER2-targeted treatment was documented in 37 of 70 patients with assessable treatment timing; exposure within the following 3 months was documented in 38 of 69 assessable patients (Supplementary Table S6).

In the reproducibility subset, baseline ICC was 0.997 (95% bootstrap interval 0.992–0.999) and follow-up ICC was 0.998 (0.996–0.999). Mean Dice was 0.904 (SD: 0.081; *n* = 30) at baseline and 0.900 (SD: 0.099; *n* = 25) at follow-up (Supplementary Table S7).

### 3.2 Local control and dose

The primary model included 102 lesions from 47 patients with 34 failures. Variational Bayesian ORs were 1.888 (95% CrI: 1.079–3.302) per unit increase in ln[1 + WT (cm^3^)], 3.071 (1.520–6.203) for edema, and 0.760 (0.697–0.829) per 10-Gy increase in BED_10_ (Figure 2). GEE estimates were weaker and less precise: 1.521 (95% CI: 0.693–3.335), 1.909 (0.822–4.434), and 0.880 (0.753–1.027), respectively (Supplementary Table S2). Same-model, same-prior MCMC yielded ORs of 1.813 (95% CrI 0.612–6.029) for WT, 3.313 (0.679–20.662) for edema, and 0.743 (0.529–0.952) for BED_10_; WT and edema intervals included 1, whereas the BED_10_ interval remained below 1. The volume-adjusted conventional BED_10_ estimate was 0.835 (95% CI: 0.696–1.002; *p* = 0.053). Exploratory spline analysis did not support a nonlinear association relative to the linear model (LR = 2.139, df = 2, *p* = 0.343); both AIC and BIC favored the linear model (*Δ*AIC = 1.861; *Δ*BIC = 7.111; Supplementary Table S3).

**Figure 2:**
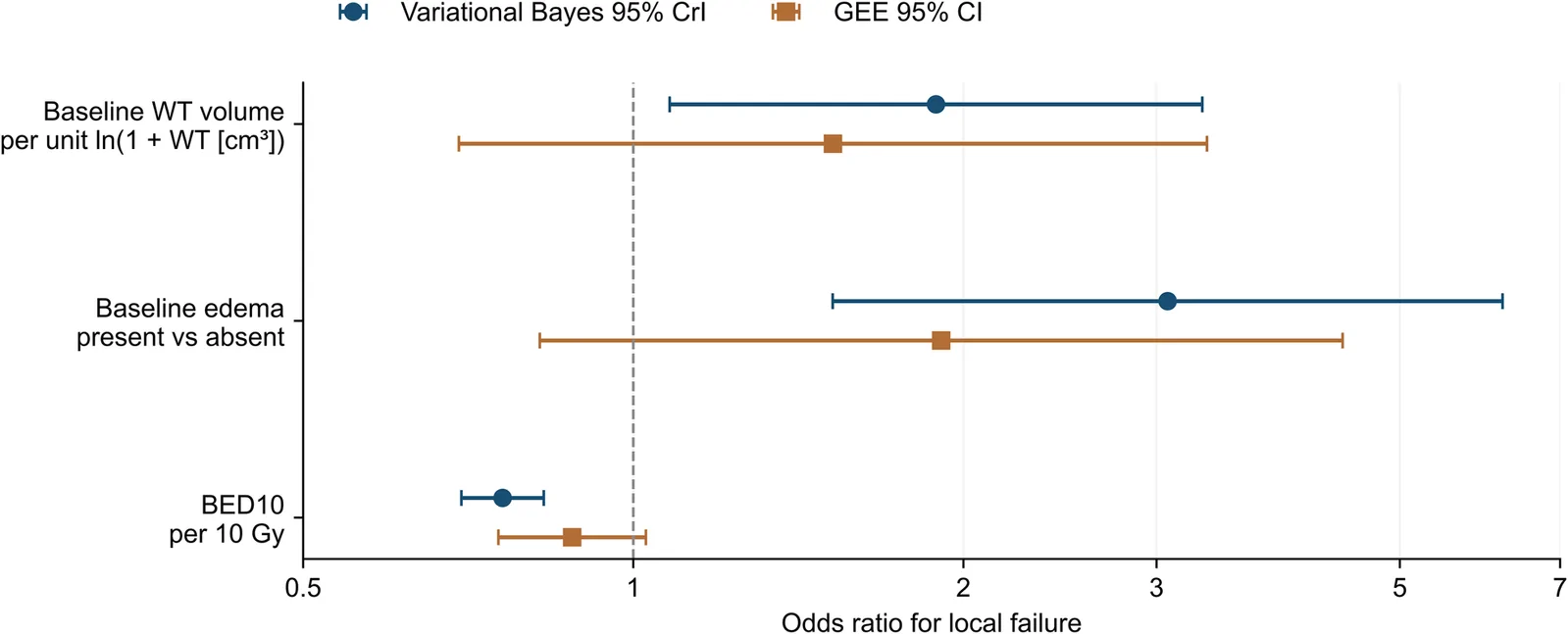
Baseline correlates of local failure in 102 lesions from 47 patients. Points and intervals show variational Bayesian mixed-effects ORs with 95% CrIs and patient-clustered GEE ORs with 95% CIs. Corresponding GEE estimates were weaker and less precise. BED_10_ is expressed per 10 Gy; the WT OR is per unit increase in ln[1 + WT (cm^3^)]. CI, confidence interval; CrI, credible interval; OR, odds ratio; WT, whole tumor.

Adjustment for molecular subtype in the same 102 lesions produced similar variational Bayesian estimates for WT, edema, and BED_10_ (Supplementary Table S2). In 99 lesions from 46 patients with 33 failures and assessable baseline HER2-targeted treatment, treatment-adjusted ORs were 1.771 (95% CrI: 1.005–3.121), 2.624 (1.286–5.356), and 0.745 (0.682–0.814), respectively. Estimates changed little relative to the base model fitted to these same 99 lesions; corresponding GEE intervals still crossed 1 (Supplementary Table S6).

### 3.3 Serial MRI response and local-control concordance

Among 154 response-evaluable lesions, 24 (15.6%) showed complete response, 56 (36.4%) partial regression, 48 (31.2%) stable volume, and 26 (16.9%) enlargement (Figure 3). Median classification time was 11.680 months (IQR: 7.483–12.649; range: 2.103–14.094). MRI was available in 138 lesions at 2–4 months, 103 at 5–7 months, and 97 at 10–14 months (Supplementary Table S5). Baseline WT volume, edema, and BED_10_ were not independently associated with enlargement in the clustered sensitivity model (Supplementary Table S3).

**Figure 3:**
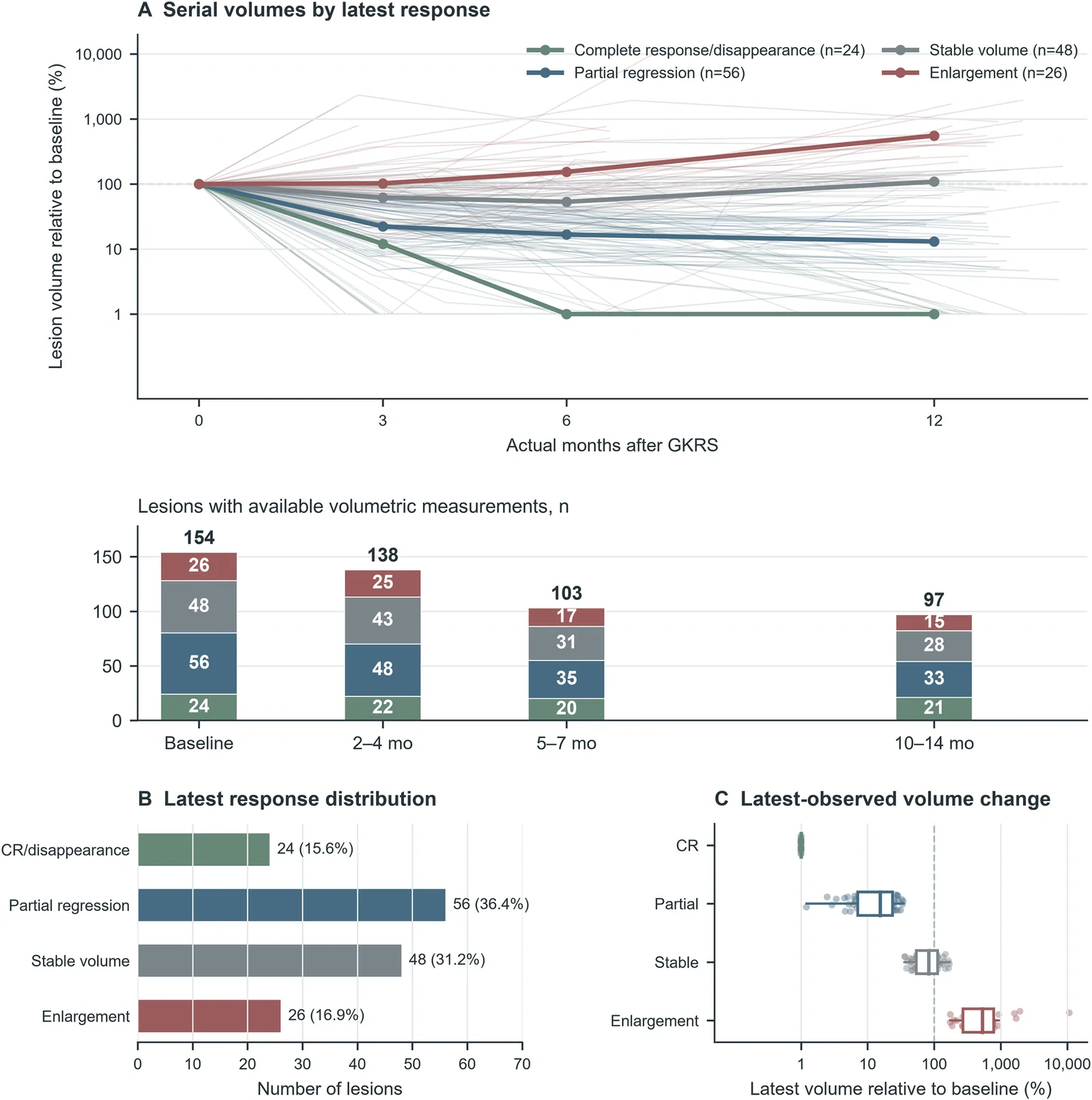
Serial WT volumes and latest-observed response. **(A)** Individual measurements at actual MRI dates (thin lines), colored by latest response category, and category-specific medians (thick lines). Window summaries use the eligible scan nearest 3, 6, or 12 months within 2–4, 5–7, or 10–14 months. The plotting cohort comprises 154 response-classifiable lesions. The stacked bars below panel A show the numbers of lesions contributing to the category-specific medians at baseline and within each follow-up window. Zero volumes are displayed at 1% on the logarithmic axis. **(B)** Response-category distribution (*n* = 154). **(C)** Latest volume relative to baseline; boxes indicate medians and interquartile ranges, and points indicate individual lesions. CR, complete response; WT, whole tumor.

Among 105 lesions with both assessments, enlargement was concordant with failure (patient-clustered OR: 16.797, 95% CI: 3.398–83.033; *p* < 0.001; Figure 4). Response assessment occurred at 10–14 months in 97 (92.4%); the other eight had earlier documented failure. Sensitivity was 54.3%, specificity 97.1%, PPV 90.5%, and NPV 81.0%. Complete concordance estimates and the same-window sensitivity analysis are reported in Supplementary Tables S4 and S5.

**Figure 4:**
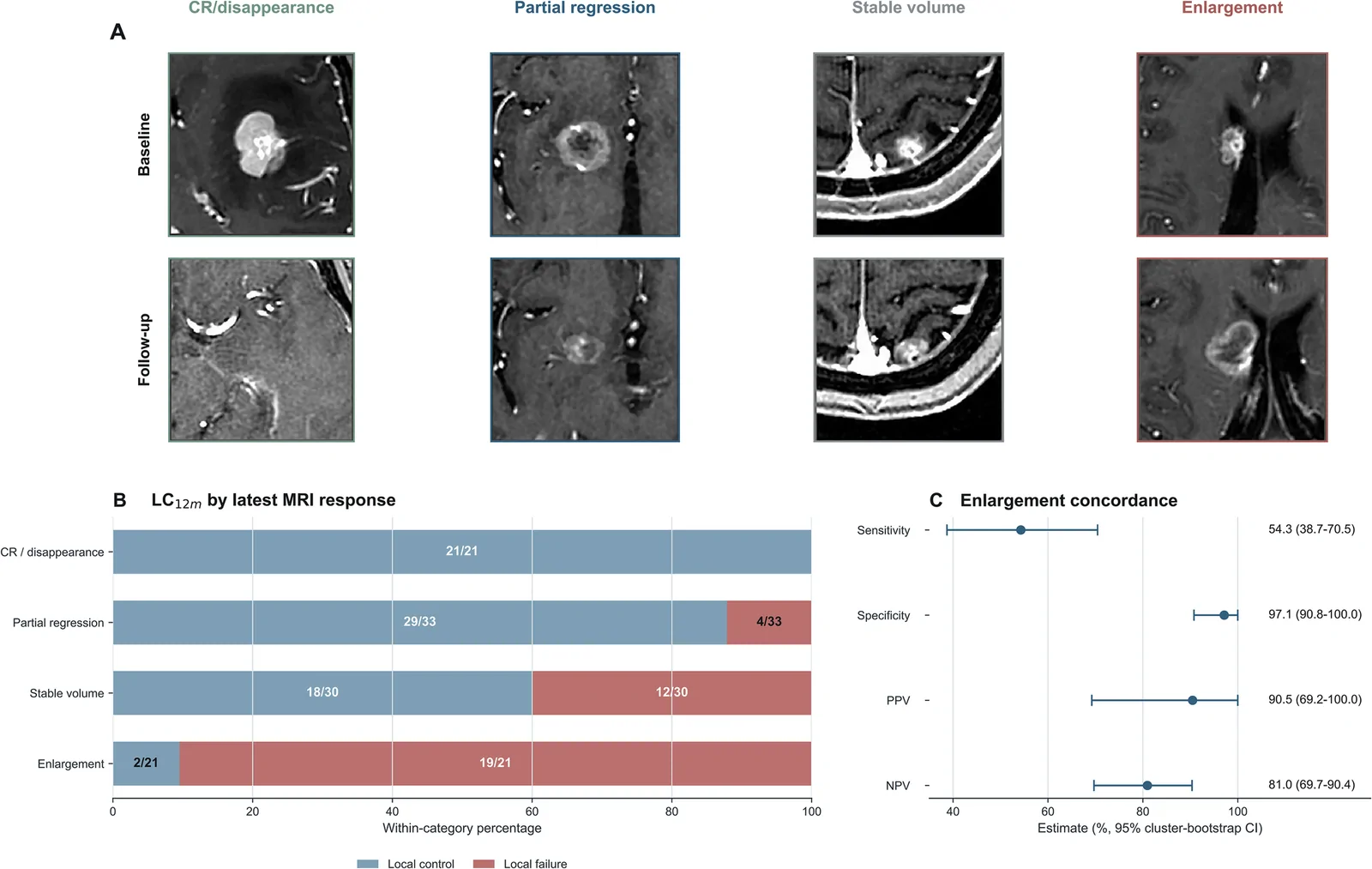
Representative MRI findings and response–outcome concordance. **(A)** Baseline and follow-up examples of disappearance, regression, stable volume, and enlargement. Follow-up was 11.24 and 7.43 months for the first two examples and the nominal 12-month visit for the latter two. **(B)** Local-control and failure proportions in 105 lesions; labels indicate counts (Cramér's V = 0.681). **(C)** Contemporaneous concordance measures for enlargement, with patient-cluster-bootstrap 95% intervals. PPV, positive predictive value; NPV, negative predictive value.

Eight lesions were adjudicated as presumed radiation injury. Six had adequate imaging for a controlled LC_12_m outcome; two remained non-evaluable. Their volume-based categories were retained independently of the clinical interpretation.

The 3- and 6-month landmark analyses included 49 and 23 lesions, with 17 and four subsequent failures. Neither included lesions meeting the enlargement threshold, preventing estimation of that contrast. The 7-month sensitivity analysis was estimable but imprecise (OR: 2.815, 95% CI: 0.295–26.890; *p* = 0.369). Alternative contrasts were also inconclusive (Supplementary Table S8).

## 4 Discussion

This study demonstrates that serial volumetric MRI can organize heterogeneous lesion-level responses after GKRS for BCBM into four clinically interpretable latest-observed response categories. The enlargement category showed strong concordance with LC_12_m and remained strongly associated with local failure after accounting for within-patient clustering and restricting assessment to the 10–14-month interval. Baseline whole-tumor volume, qualitative edema, and BED_10_ provided complementary information on local-failure associations in the variational fit of the prespecified Bayesian model. Together, these findings link baseline imaging characteristics, biological dose, and post-treatment MRI evolution within a descriptive lesion-level response-assessment framework.

The association between larger baseline whole-tumor volume and local failure in the primary variational Bayesian model is consistent with breast-cancer-specific and broader SRS literature (13, 14, 18, 19). Larger tumors may contain more heterogeneous viable and necrotic tissue, present greater planning complexity, and receive lower prescription doses because of toxicity constraints. Qualitative edema may similarly identify a more adverse local imaging phenotype, although the present study cannot establish whether edema is a causal biological driver. It may reflect blood-brain-barrier disruption, vascular permeability, inflammation, or lesion size. A recent systematic review and meta-analysis likewise found pretreatment perilesional edema to be associated with a higher risk of local failure after SRS for brain metastases, including studies using a binary assessment of edema (29). Estimates for both variables were directionally consistent across variational Bayesian, MCMC, and GEE analyses, although the MCMC and GEE intervals included 1. These findings support considering baseline whole-tumor volume and edema as candidate imaging correlates for contextualizing lesion-level response after GKRS.

Higher BED_10_ remained inversely associated with local failure in the variational fit of the prespecified Bayesian mixed-effects model, but the magnitude and precision of this association depended on model specification. The unadjusted dose difference was substantial, whereas the same-covariate GEE and conventional volume-adjusted linear model yielded attenuated estimates. Same-model, same-prior MCMC retained the inverse BED_10_ association, with a wider interval than the variational estimate. Differences in estimands and priors may contribute to the contrast with GEE, whereas differences between variational estimation and MCMC concern posterior approximation under the same model. This pattern is clinically plausible because dose prescription is confounded by lesion size and anatomical feasibility, and established dose-selection studies emphasize the interaction between target size, fractionation, and toxicity (17–19). Additional adjustment for molecular subtype or HER2-targeted treatment at GKRS changed the estimates little relative to the corresponding base models. Exploratory restricted cubic spline analysis did not significantly improve fit over the linear model (*p* = 0.343), providing no evidence for a nonlinear dose–response relationship within this cohort. The results therefore support reporting biological dose together with lesion burden while avoiding a universal escalation recommendation from this cohort (26, 27). Preliminary prospective data from the SAFER study also support a dose-response relationship between BED10 and local control, although its dose model, follow-up horizon, and study population differ from ours (30). Specifically, SAFER used prescription doses and the linear–quadratic–cubic model, whereas our study used mean target doses over the initial treatment course and the linear–quadratic model, limiting direct comparison of numerical BED_10_ values.

The serial volumetric analysis is the most distinctive element of this study. Binary local control remains important for comparative outcome assessment, but it compresses a temporally heterogeneous process. The four volumetric response categories preserved clinically meaningful differences among lesion disappearance, regression, stability, and enlargement at the latest eligible assessment relative to baseline. Serial plots additionally retain intermediate changes that are not captured by the latest category alone. The strong association between the enlargement category and LC_12_m was maintained after patient-level clustering and after restriction to lesions classified within the 10–14-month interval, supporting its concordance with local failure. Its high specificity and positive predictive value, alongside moderate sensitivity, indicate that enlargement frequently accompanied local failure, whereas absence of enlargement did not exclude failure. Because the category and endpoint share volumetric information and were predominantly assessed contemporaneously, this association does not establish independent predictive value. The response framework is intended to complement formal local-control assessment by describing the magnitude and direction of individual lesion changes, particularly when post-SRS enlargement may reflect tumor progression, radiation injury, or mixed treatment effects (23, 32). No eligible lesions met the enlargement threshold in the 3- or 6-month landmark analyses, and the extended-window estimates were imprecise. Previous work found that a ≥20% volume reduction at 3 months, but not a ≥65% reduction, distinguished subsequent local control among lesions without progression at that time (28). This suggests that the prognostic information provided by early volumetric response may depend on threshold selection and warrants further evaluation. Recent longitudinal brain-metastasis imaging resources similarly demonstrate the value of serially annotated MRI for standardized disease assessment and method development (31). Prospective studies using standardized assessment intervals and repeated volumetric measurements may further establish the clinical value of this framework.

This retrospective single-center study had limited local-failure events, with 34 failures in the primary model, and LC_12_m was evaluable in 105 of 182 lesions, creating potential follow-up selection bias. Response categories were based on latest-observed MRI, and unequal follow-up affected the opportunity to detect late progression. Radiation injury was adjudicated using clinical and serial imaging findings without histopathologic confirmation. Molecular subtype was available in 79 patients, and baseline HER2-targeted treatment was included in sensitivity analysis; other systemic treatments and subsequent treatment changes may still contribute to residual confounding. Segmentation reproducibility was assessed in a 30-lesion subset, and small-lesion contour variability remains relevant near percentage-change thresholds. Baseline and follow-up MRI differed in acquisition slice thickness. External validation across SRS platforms using standardized follow-up and reproducible segmentation remains necessary.

## 5 Conclusions

Serial volumetric MRI and latest-observed response categories provide a clinically interpretable description of lesion-level response after GKRS for BCBM, complementing binary local-control assessment. The enlargement category showed strong concordance with LC_12_m, although shared volumetric information and overlapping assessment times limit claims of independent prediction. Baseline WT volume, edema, and BED_10_ were associated with failure in the variational Bayesian model, with model-dependent statistical support. External validation with standardized follow-up and reproducible segmentation should assess the added clinical value of this framework and improve differentiation of tumor progression from treatment effect.

## Data Availability

The original contributions presented in the study are included in the article/Supplementary Material, further inquiries can be directed to the corresponding author.
