# Supplementary material for: Latest observed volumetric MRI response and lesion level local control after gamma knife radiosurgery for breast cancer brain metastases
Source: Front Med (Lausanne). 2026 Oct 28;13:1998918. doi: 10.3389/fmed.2026.1998918 (PMC13752028; doi:10.3389/fmed.2026.1998918)
Supplement: Supplementary file 1 [file Table1.docx]

**Supplementary Materials**

Latest Observed Volumetric MRI Response and Lesion Level Local Control after Gamma Knife Radiosurgery for Breast Cancer Brain Metastases

Sijun Diao^a^^†^, Tao Huang^a†^, Zhongyu Li^a^, Shihong Zhu^a^, Fujun Liu ^a^, Xiyue Lu^b^, Huiyang Luo^b^, Jing Chen^a*^

a. Department of Neurosurgery, West China Hospital, West China Medical School, Sichuan University, Chengdu 610041, China

b. Department of Anesthesiology, West China Hospital, West China Medical School, Sichuan University, Chengdu 610041, China

^†^These authors contributed equally to this work

^*^Correspondence: Jing Chen, [chenjing6811@126.com](mailto:chenjing6811@126.com)

ORCID: Jing Chen, 0000-0002-3297-9183

**Table S1. Reasons for non-evaluable 12-month local control and comparison of evaluable and non-evaluable lesions**

**A. Reasons for non-evaluable LC₁₂m**

| **Reason** | **n (%) of 77 lesions** |
| --- | --- |
| Death before 10 months without documented earlier failure | 47 (61.0%) |
| Death during 10–14 months without a qualifying MRI | 4 (5.2%) |
| No qualifying 10–14-month MRI in available records | 20 (26.0%) |
| Unresolved tumor progression versus treatment effect | 3 (3.9%) |
| Inadequate contrast MRI or usable volumetry | 2 (2.6%) |
| Presumed radiation injury; inadequate window assessment | 1 (1.3%) |

**B. Baseline and treatment characteristics by LC₁₂m evaluability**

| **Characteristic** | **Evaluable n** | **Evaluable summary** | **Non-evaluable n** | **Non-evaluable summary** | **SMD** |
| --- | --- | --- | --- | --- | --- |
| Baseline WT volume, cm³ | 105 | 0.451 (0.174–1.424) | 77 | 0.319 (0.101–1.318) | -0.047 |
| Baseline ET volume, cm³ | 105 | 0.391 (0.148–1.297) | 64 | 0.309 (0.103–1.305) | 0.013 |
| First-session margin dose, Gy | 105 | 13.000 (12.000–18.000) | 77 | 13.000 (12.000–13.000) | 0.475 |
| Prescription isodose line, % | 105 | 45.000 (45.000–50.000) | 77 | 50.000 (45.000–50.000) | -0.050 |
| Age at GKRS, years | 105 | 51.000 (43.000–59.000) | 77 | 55.000 (45.000–59.000) | 0.031 |
| KPS | 103 | 90.000 (90.000–100.000) | 27 | 90.000 (80.000–100.000) | 0.040 |
| Baseline brain metastasis count | 105 | 4.000 (2.000–5.000) | 77 | 4.000 (2.000–5.000) | -0.202 |
| Qualitative edema | 105 | 67/105 (63.8%) | 39 | 12/39 (30.8%) | 0.701 |
| Intracranial hemorrhage | 103 | 6/103 (5.8%) | 27 | 1/27 (3.7%) | 0.100 |
| Location: supratentorial | 103 | 68/103 (66.0%) | 27 | 20/27 (74.1%) | -0.177 |
| Location: infratentorial | 103 | 34/103 (33.0%) | 27 | 7/27 (25.9%) | 0.156 |
| Location: brainstem | 103 | 1/103 (1.0%) | 27 | 0/27 (0.0%) | 0.140 |
| Subtype: HER2-positive | 105 | 53/105 (50.5%) | 74 | 26/74 (35.1%) | 0.314 |
| Subtype: Luminal B | 105 | 34/105 (32.4%) | 74 | 28/74 (37.8%) | -0.115 |
| Subtype: Luminal A | 105 | 0/105 (0.0%) | 74 | 7/74 (9.5%) | -0.457 |
| Subtype: triple-negative breast cancer | 105 | 18/105 (17.1%) | 74 | 13/74 (17.6%) | -0.011 |
| HR positive | 99 | 33/99 (33.3%) | 23 | 10/23 (43.5%) | -0.210 |
| HER2 IHC 3+ | 99 | 59/99 (59.6%) | 23 | 12/23 (52.2%) | 0.150 |
| Extracranial metastasis | 103 | 82/103 (79.6%) | 27 | 23/27 (85.2%) | -0.147 |
| Prior WBRT | 103 | 33/103 (32.0%) | 27 | 6/27 (22.2%) | 0.222 |
| Prior intracranial surgery | 103 | 35/103 (34.0%) | 27 | 3/27 (11.1%) | 0.569 |

Values are medians (IQR) or n/N (%), using available observations in each group. SMD compares evaluable minus non-evaluable groups, standardized by the pooled standard deviation or binary variance; it is descriptive rather than a significance test. Patient-level features are repeated across that patient’s lesions in this lesion-level comparison. Death-associated categories are based on the timing of death relative to MRI availability. Missing outcomes were not imputed. IQR, interquartile range; SMD, standardized mean difference; WT, whole tumor; ET, enhancing tumor; GKRS, Gamma Knife radiosurgery; LC₁₂m, 12-month local control; KPS, Karnofsky Performance Status; HR, hormone receptor; HER2, human epidermal growth factor receptor 2; IHC, immunohistochemistry; WBRT, whole-brain radiotherapy.

**Table S2. Prespecified Bayesian mixed-effects model and same-covariate patient-clustered GEE sensitivity analysis for local failure**

**A. Prespecified model and same-covariate sensitivity analysis**

| **Variable** | **Bayesian OR (95% CrI)** | **GEE OR (95% CI)** | **GEE p value** |
| --- | --- | --- | --- |
| ln[1 + baseline WT (cm³)] | 1.888 (1.079–3.302) | 1.521 (0.693–3.335) | 0.295 |
| Qualitative edema | 3.071 (1.520–6.203) | 1.909 (0.822–4.434) | 0.133 |
| BED₁₀ per 10 Gy | 0.760 (0.697–0.829) | 0.880 (0.753–1.027) | 0.104 |

Both models included 102 lesions from 47 patients with 34 failures; three outcome-evaluable lesions lacked BED₁₀. Analyses used Python and statsmodels 0.14.5. BinomialBayesMixedGLM used a patient random intercept, mean-field variational Bayes, BFGS optimization, and up to 2,000 iterations. Fixed-effect priors were independent zero-mean Gaussians (SD 2); log standard deviations of variance components had zero-mean Gaussian priors (SD 1). GEE used exchangeable correlation and patient-robust covariance. All GEE intervals crossed 1, whereas the three variational Bayesian intervals excluded 1. Estimates were consistent across three converged initializations, with no convergence warnings. A same-prior non-centered Hamiltonian Monte Carlo sensitivity analysis used four chains, each with 2,500 warmup iterations and 8,000 retained draws. Posterior median ORs (95% CrI) were 1.813 (0.612–6.029) for WT, 3.313 (0.679–20.662) for edema, and 0.743 (0.529–0.952) for BED₁₀ per 10 Gy. The maximum split R-hat was 1.001 and the minimum effective sample size was 4,438; no divergence flags were recorded. The random-intercept SD was 2.832 (95% CrI 1.270–5.962).

**B. Molecular-subtype-adjusted sensitivity analysis**

| **Variable** | **Subtype-adjusted VB OR (95% CrI)** | **Subtype-adjusted GEE OR (95% CI)** | **GEE p** |
| --- | --- | --- | --- |
| ln[1 + WT (cm³)] | 1.897 (1.074–3.351) | 1.512 (0.664–3.443) | 0.325 |
| Qualitative edema | 3.206 (1.562–6.582) | 1.907 (0.822–4.425) | 0.133 |
| BED₁₀ per 10 Gy | 0.740 (0.678–0.809) | 0.875 (0.751–1.020) | 0.087 |
| Luminal B vs HER2-positive | 1.711 (0.608–4.813) | 1.383 (0.405–4.715) | 0.605 |
| Triple-negative vs HER2-positive | 1.007 (0.229–4.436) | 1.101 (0.302–4.014) | 0.884 |

The subtype-adjusted sensitivity analysis included the same 102 lesions, 47 patients, and 34 failures as the primary model. The four recorded subtype categories were retained; no Luminal A patient contributed a model lesion, so its coefficient was not estimable. HER2-positive was the reference category. Priors, variational estimation, and GEE specifications were unchanged. VB, variational Bayes; GEE, generalized estimating equations; OR, odds ratio; CrI, credible interval; CI, confidence interval; SD, standard deviation; WT, whole tumor; BED₁₀, biologically effective dose with α/β = 10 Gy; BFGS, Broyden–Fletcher–Goldfarb–Shanno.

**Table S3. Sensitivity analyses of BED₁₀ associations with local failure and latest-observed response**

**A. BED₁₀ distribution by local-control status**

| **Local-control status** | **n** | **BED₁₀, median (IQR), Gy** |
| --- | --- | --- |
| Local control | 68 | 72.078 (49.198–111.480) |
| Local failure | 34 | 48.735 (29.696–67.551) |

Two-sided Mann–Whitney U = 1664; p < 0.001. This unclustered comparison is descriptive.

**B. Linear BED₁₀ model adjusted for baseline WT volume**

| **Term** | **OR (95% CI)** | **p value** | **n lesions** | **n patients** |
| --- | --- | --- | --- | --- |
| BED₁₀ per 10 Gy | 0.835 (0.696–1.002) | 0.053 | 102 | 47 |
| ln[1 + WT (cm³)] | 1.959 (0.865–4.438) | 0.107 | 102 | 47 |

Linear and spline models included 102 lesions. Linear-model covariance was patient-cluster-robust.

**C. Linear versus restricted cubic spline model comparison**

| **Exposure** | **LR statistic** | **df** | **p value** | **ΔAIC** | **ΔBIC** |
| --- | --- | --- | --- | --- | --- |
| BED₁₀ | 2.139 | 2 | 0.343 | 1.861 | 7.111 |
| EQD2 | 2.139 | 2 | 0.343 | 1.861 | 7.111 |

ΔAIC and ΔBIC are spline minus linear; positive values favor the linear model. Likelihood-ratio tests compared ordinary model likelihoods and were descriptive, without adjustment for patient clustering. EQD2 = BED₁₀/1.2 is a linear rescaling.

**D. Restricted cubic spline knot locations**

| **Exposure** | **5th percentile, Gy** | **35th percentile, Gy** | **65th percentile, Gy** | **95th percentile, Gy** |
| --- | --- | --- | --- | --- |
| BED₁₀ | 28.467 | 49.606 | 77.319 | 141.455 |
| EQD2 | 23.723 | 41.338 | 64.432 | 117.879 |

Splines used boundary knots at the 5th/95th and internal knots at the 35th/65th percentiles.

**E. BED₁₀ comparison across four response categories**

| **n lesions** | **Permutation statistic** | **p value** | **Permutations** |
| --- | --- | --- | --- |
| 150 | 6.016 | 0.111 | 10,000 |

The descriptive dose permutation used 150 lesions and 10,000 unrestricted permutations, without accounting for patient clustering.

**F. Patient-clustered GEE for enlargement versus other response categories**

| **Term** | **OR (95% CI)** | **p value** | **n lesions** | **n patients** |
| --- | --- | --- | --- | --- |
| Peritumoral edema | 1.517 (0.572–4.027) | 0.402 | 140 | 63 |
| ln[1 + WT (cm³)] | 0.545 (0.189–1.573) | 0.262 | 140 | 63 |
| BED₁₀ per 10 Gy | 0.959 (0.819–1.122) | 0.598 | 140 | 63 |

Enlargement GEE used 140 complete cases from 63 patients with exchangeable correlation. BED₁₀, biologically effective dose with α/β = 10 Gy; EQD2, equivalent dose in 2-Gy fractions; WT, whole tumor; GEE, generalized estimating equations; OR, odds ratio; CI, confidence interval; IQR, interquartile range; LR, likelihood ratio; df, degrees of freedom; AIC, Akaike information criterion; BIC, Bayesian information criterion.

**Table S4. Association between enlargement response category and local failure: patient-clustered GEE and cluster-bootstrap analyses**

**A. Patient-clustered GEE**

| **Comparison** | **Lesions / patients** | **Failures / enlarged lesions** | **OR (95% CI)** | **p value** |
| --- | --- | --- | --- | --- |
| Enlargement vs other categories | 105 / 47 | 35 / 21 | 16.797 (3.398–83.033) | <0.001 |

**B. Patient-cluster bootstrap (5,000 resamples)**

| **Metric** | **Point estimate** | **Bootstrap median** | **95% interval** | **Valid replicates** | **Total replicates** |
| --- | --- | --- | --- | --- | --- |
| Cramers V | 0.681 | 0.692 | 0.542–0.810 | 5000 | 5000 |
| Sensitivity | 0.543 | 0.542 | 0.387–0.705 | 5000 | 5000 |
| Specificity | 0.971 | 0.972 | 0.908–1.000 | 5000 | 5000 |
| PPV | 0.905 | 0.909 | 0.692–1.000 | 5000 | 5000 |
| NPV | 0.810 | 0.812 | 0.697–0.904 | 5000 | 5000 |

GEE used a binomial family with exchangeable patient correlation. Bootstrap resampled patients with all their lesions (5,000 replicates). Response-by-outcome counts were CR 21/0, regression 29/4, stable volume 18/12, and enlargement 2/19 (control/failure). Pearson χ² = 48.639 and Cramér’s V = 0.681; a descriptive unrestricted lesion-level permutation gave p < 0.001 (20,000 permutations). These metrics quantify contemporaneous concordance. CR, complete response; GEE, generalized estimating equations; PPV, positive predictive value; NPV, negative predictive value; OR, odds ratio; CI, confidence interval.

**Table S5. Timing of latest-observed response classification and 10–14-month sensitivity analysis**

**A. All 154 response-evaluable lesions**

| **Item** | **Value** |
| --- | --- |
| Classification interval, months | Median 11.680; IQR 7.483–12.649; range 2.103–14.094 |
| ≤6 months, n | 26 |
| >6 to <10 months, n | 28 |
| 10–14 months, n | 97 |
| >14 months, n | 3 |
| Post-local-salvage measurements used for classification, n | 0 |
| Dated follow-up WT measurements, n | 393 |

**B. Lesions with both response category and LC₁₂m**

| **Item** | **Value** |
| --- | --- |
| Lesions / patients / failures | 105 / 47 / 35 |
| Classification interval, months | Median 12.287; IQR 11.532–12.846; range 2.267–13.569 |
| ≤6 months, n | 3 |
| >6 to <10 months, n | 5 |
| 10–14 months, n | 97 |
| >14 months, n | 0 |

**C. Classification interval by category in the 105-lesion overlap set**

| **Category** | **n** | **Median, months** | **IQR, months** | **Range, months** | **10–14 months, n (%)** |
| --- | --- | --- | --- | --- | --- |
| CR / disappearance | 21 | 12.550 | 12.222–12.846 | 11.400–13.569 | 21 (100.0%) |
| Partial regression | 33 | 12.222 | 11.335–12.846 | 11.236–13.536 | 33 (100.0%) |
| Stable volume | 30 | 12.140 | 11.400–12.838 | 2.267–13.405 | 28 (93.3%) |
| Enlargement | 21 | 12.386 | 7.819–12.879 | 2.267–13.437 | 15 (71.4%) |

**D. Patient-clustered GEE restricted to classifications at 10–14 months**

| **n lesions** | **n patients** | **Local failures** | **OR (95% CI)** | **p value** |
| --- | --- | --- | --- | --- |
| 97 | 42 | 27 | 17.716 (2.728–115.059) | 0.003 |

Time was measured from index GKRS using 30.4375 days per month. Classification used the latest eligible pre-salvage WT volume. Of 105 jointly evaluable lesions, 97 (92.4%) were classified at 10–14 months and eight earlier, with established failure. Nominal 3-, 6-, and 12-month records numbered 146, 133, and 112; actual 2–4-, 5–7-, and 10–14-month counts were 138, 103, and 97. Two additional observations were obtained after GKRS and before salvage surgery, contributing to 393 dated follow-up lesion-volume measurements. Classification times were determined by the available eligible examinations rather than a strict 14-month cutoff. Follow-up data collection ended in August 2026. GKRS, Gamma Knife radiosurgery; WT, whole tumor; LC₁₂m, 12-month local control; IQR, interquartile range; GEE, generalized estimating equations; OR, odds ratio; CI, confidence interval.

**Table S6. Systemic therapy at GKRS and within 3 months afterward, and baseline HER2-targeted treatment adjustment**

**A. Systemic treatment exposure**

| **Therapy class** | **At GKRS, n/N (%)** | **Within 3 months after GKRS, n/N (%)** |
| --- | --- | --- |
| Chemotherapy | 35/70 (50.0%) | 41/73 (56.2%) |
| HER2-targeted therapy | 37/70 (52.9%) | 38/69 (55.1%) |
| Endocrine therapy | 7/70 (10.0%) | 7/69 (10.1%) |
| CDK4/6 inhibitor | 2/70 (2.9%) | 2/69 (2.9%) |
| Antibody–drug conjugate | 16/70 (22.9%) | 17/69 (24.6%) |
| Immunotherapy | 2/70 (2.9%) | 2/69 (2.9%) |

Treatment exposure was determined from documented drug regimens and start/end or observation dates in 80 patients. Categories were not mutually exclusive. At-GKRS exposure required a recorded treatment interval covering the index date. The subsequent window extended from GKRS to 3 calendar months afterward and captured any documented exposure. Non-exposure was assigned only when the documented complete regimen for the relevant date or window contained no drug from the specified class, or when an explicit no-treatment record was available; otherwise, exposure status was coded as unknown. Denominators vary by class and window. Antibody–drug conjugates targeting HER2 contribute to both categories.

**B. Baseline HER2-targeted treatment adjustment**

| **Variable** | **Base-model VB OR (95% CrI)** | **HER2-treatment-adjusted VB OR (95% CrI)** | **Base-model GEE OR (95% CI)** | **HER2-treatment-adjusted GEE OR (95% CI)** |
| --- | --- | --- | --- | --- |
| ln[1 + WT (cm³)] | 1.825 (1.042–3.197) | 1.771 (1.005–3.121) | 1.516 (0.673–3.419) | 1.484 (0.656–3.360) |
| Qualitative edema | 2.844 (1.407–5.747) | 2.624 (1.286–5.356) | 1.858 (0.797–4.328) | 1.740 (0.753–4.022) |
| BED₁₀ per 10 Gy | 0.764 (0.701–0.833) | 0.745 (0.682–0.814) | 0.878 (0.751–1.027) | 0.871 (0.741–1.024) |
| HER2-targeted therapy at GKRS | Not included | 2.002 (0.931–4.302) | Not included | 1.655 (0.527–5.199) |

The treatment-adjusted sensitivity analysis included 99 lesions from 46 patients with 33 failures. Three primary-model lesions lacked assessable baseline HER2-targeted exposure and were excluded from both models in this comparison. Exposure was matched to each lesion’s index GKRS date. The base model and HER2-treatment-adjusted model both included WT, edema, and BED₁₀ and were fitted to the same 99 lesions using variational Bayes and patient-clustered GEE. Patient clustering and the original priors were retained. The treatment-adjusted model additionally included HER2-targeted therapy at GKRS; post-GKRS treatment was not treated as a baseline exposure. Treatment-associated ORs do not estimate causal drug efficacy. VB, variational Bayes; GEE, generalized estimating equations; OR, odds ratio; CrI, credible interval; CI, confidence interval.

**Table S7. Interobserver WT segmentation reproducibility**

| **Time point** | **Volume pairs** | **ICC(A,1) and 95% interval** | **Dice pairs** | **Mean Dice (SD)** | **Dice median (IQR)** |
| --- | --- | --- | --- | --- | --- |
| Baseline | 30 | 0.997 (0.992–0.999) | 30 | 0.904 (0.081) | 0.909 (0.874–0.962) |
| Follow-up | 30 | 0.998 (0.996–0.999) | 25 | 0.900 (0.099) | 0.941 (0.883–0.965) |

The availability-limited subset comprised 30 lesions from 30 patients. Constrained stratified sampling selected 10 lesions in each baseline-volume stratum (cutpoints 0.192390 and 1.163042 cm³) and response-category quotas of 5 disappearance, 11 regression, 9 stable, and 5 enlargement lesions. Paired independent WT masks were used for nonzero measurements; five disappearances were independently confirmed by both readers, each recording a volume of zero; no mask files were created for these follow-up observations. ICC(A,1) is a two-way mixed-effects absolute-agreement single-measure coefficient. Patient-level percentile bootstrap intervals used 20,000 replicates and unrounded reader-specific volumes. Five disappearances independently confirmed by both readers contributed paired zero volumes to ICC. Dice was not calculated for these five follow-up observations because neither reader identified a visible lesion and no masks were available. Nonzero-only follow-up ICC was 0.998 (95% interval 0.996–0.999; n = 25). Dice ranged from 0.622 to 0.989 at baseline and 0.667 to 0.996 at follow-up. WT, whole tumor; ICC, intraclass correlation coefficient; SD, standard deviation; IQR, interquartile range.

**Table S8. Early landmark analyses**

**A. Landmark cohorts and subsequent outcomes**

| **Landmark** | **MRI window** | **Lesions / patients** | **Subsequent failures** | **CR / PR / stable / enlarged** | **Failures among enlarged** |
| --- | --- | --- | --- | --- | --- |
| 3 months | 2–3 months | 49 / 21 | 17 | 7 / 21 / 21 / 0 | 0 / 0 |
| 6 months | 5–6 months | 23 / 11 | 4 | 5 / 9 / 9 / 0 | 0 / 0 |
| 4 months | 2–4 months | 95 / 41 | 27 | 10 / 37 / 44 / 4 | 0 / 4 |
| 7 months | 5–7 months | 67 / 30 | 7 | 12 / 29 / 21 / 5 | 1 / 5 |

Eligibility required survival and absence of local failure or lesion-directed salvage at the landmark, with an outcome assessment afterward. Outcomes used the LC₁₂m definition. Using 30.4375 days per month, scans nearest 3 or 6 months were selected within the specified pre-landmark window. Wider MRI windows of 2–4 and 5–7 months were paired with landmarks at 4 and 7 months. Contrasts compared enlargement with other categories, and stable/enlarged lesions with CR/regression. GEE contrasts with no exposure variation or an empty 2×2 cell were designated non-estimable.

**B. Cohort selection from all 182 lesions**

| **Status** | **3 months** | **6 months** | **4 months** | **7 months** |
| --- | --- | --- | --- | --- |
| Death by landmark | 8 | 31 | 21 | 34 |
| Failure by landmark | 6 | 10 | 6 | 21 |
| Local salvage by landmark | 0 | 0 | 0 | 0 |
| No eligible pre-landmark MRI | 97 | 116 | 34 | 47 |
| Subsequent outcome unavailable | 22 | 2 | 26 | 13 |
| No later outcome assessment | 0 | 0 | 0 | 0 |
| Included | 49 | 23 | 95 | 67 |

Exclusions were applied sequentially in the order shown and were mutually exclusive.

**C. Patient-clustered GEE contrasts**

| **Landmark** | **Contrast** | **n / failures** | **OR (95% CI)** | **p value** |
| --- | --- | --- | --- | --- |
| 3 months | Enlargement vs other | 49 / 17 | NE | NA |
| 3 months | Stable/enlarged vs CR/PR | 49 / 17 | 0.766 (0.242–2.422) | 0.650 |
| 6 months | Enlargement vs other | 23 / 4 | NE | NA |
| 6 months | Stable/enlarged vs CR/PR | 23 / 4 | 4.723 (0.375–59.471) | 0.230 |
| 4 months | Enlargement vs other | 95 / 27 | NE | NA |
| 4 months | Stable/enlarged vs CR/PR | 95 / 27 | 1.095 (0.511–2.348) | 0.816 |
| 7 months | Enlargement vs other | 67 / 7 | 2.815 (0.295–26.890) | 0.369 |
| 7 months | Stable/enlarged vs CR/PR | 67 / 7 | 2.109 (0.577–7.709) | 0.259 |

NE denotes non-estimable because a 2×2 cell was empty or exposure did not vary; NA, not applicable. The estimable 7-month enlargement contrast gave OR 2.815 (95% CI 0.295–26.890; p = 0.369). The 4-month stable/enlarged contrast adjusted for baseline WT, edema, and BED₁₀ included 92 lesions with 26 failures: OR 1.131 (0.414–3.092; p = 0.810). Covariate-adjusted analyses were restricted to contrasts with at least 50 lesions and 20 failures. GKRS, Gamma Knife radiosurgery; LC₁₂m, 12-month local control; CR, complete response; PR, partial regression; GEE, generalized estimating equations; WT, whole tumor; BED₁₀, biologically effective dose with α/β = 10 Gy; OR, odds ratio; CI, confidence interval.

**Table S9. Fractionation and mean target dose parameters used to calculate BED₁₀**

**A. Complete-case primary model (102 lesions)**

| **n** | **Lesion N** | **Total mean target dose D (Gy)** | **Mean target dose per fraction D/n (Gy)** | **BED₁₀ (Gy)** |
| --- | --- | --- | --- | --- |
| 1 | 67 | 23.873 (20.788–29.442) | 23.873 (20.788–29.442) | 80.867 (64.002–116.125) |
| 2 | 35 | 18.486 (16.209–19.730) | 9.243 (8.105–9.865) | 35.572 (29.348–39.193) |

**B. All lesions with available dose (178 lesions)**

| **n** | **Lesions N** | **Total mean target dose D (Gy)** | **Mean target dose per fraction D/n (Gy)** | **BED₁₀ (Gy)** |
| --- | --- | --- | --- | --- |
| 1 | 132 | 21.680 (18.313–26.924) | 21.680 (18.313–26.924) | 68.680 (51.848–99.417) |
| 2 | 46 | 18.524 (16.999–20.192) | 9.262 (8.500–10.096) | 35.681 (31.448–40.577) |

Dose parameters are grouped by the number of fractions. Continuous values are medians (interquartile ranges).

D is the total mean target dose over the initial course, n is the number of fractions obtained from treatment records, and D/n is the mean target dose per fraction. Two-fraction courses comprised two equal fractions. BED₁₀ = D[1 + D/(10n)], with α/β = 10 Gy. Subsequent lesion-directed salvage irradiation was not included. The cohort comprised 136 single-fraction and 46 two-fraction lesions. Four single-fraction lesions lacked usable dose values, leaving 178 for panel B. Panel A comprises the 102 complete cases used in the Bayesian and GEE local-failure models. Dose values represent mean target doses rather than marginal prescription doses.
